# Specific PKC isoforms regulate LPS-stimulated iNOS induction in murine microglial cells

**DOI:** 10.1186/1742-2094-8-38

**Published:** 2011-04-21

**Authors:** Jie Wen, Rachel Ribeiro, Yumin Zhang

**Affiliations:** 1 Department of Anatomy, Physiology and Genetics, Uniformed Services University of the Health Sciences, 4301 Jones Bridge Road, Bethesda, MD 20814, USA; 2 Program in Neuroscience, Uniformed Services University of the Health Sciences, 4301 Jones Bridge Road, Bethesda, MD 20814, USA

## Abstract

**Background:**

Excessive production of nitric oxide (NO) by inducible nitric oxide synthase (iNOS) in reactive microglia is a major contributor to initiation/exacerbation of inflammatory and degenerative neurological diseases. Previous studies have indicated that activation of protein kinase C (PKC) can lead to iNOS induction. Because of the existence of various PKC isoforms and the ambiguous specificity of PKC inhibitors, it is unclear whether all PKC isoforms or a specific subset are involved in the expression of iNOS by reactive microglia. In this study, we employed molecular approaches to characterize the role of each specific PKC isoform in the regulation of iNOS expression in murine microglia.

**Methods:**

Induction of iNOS in response to bacterial endotoxin lipopolysaccharide (LPS) was measured in BV-2 murine microglia treated with class-specific PKC inhibitors, or transfected with siRNA to silence specific PKC isoforms. iNOS expression and MAPK phosphorylation were evaluated by western blot. The role of NF-κB in activated microglia was examined by determining NF-κB transcriptional response element- (TRE-) driven, promoter-mediated luciferase activity.

**Results:**

Murine microglia expressed high levels of nPKCs, and expressed relatively low levels of cPKCs and aPKCs. All PKC inhibitors attenuated induction of iNOS in LPS-activated microglia. Knockdown of PKC δ and PKC β attenuated ERK1/2 and p38 phosphorylation, respectively, and blocked NF-κB activation that leads to the expression of iNOS in reactive microglia.

**Conclusions:**

Our results identify PKC δ and β as the major PKC isoforms regulating iNOS expression in reactive microglia. The signaling pathways mediated by PKC involve phosphorylation of distinct MAPKs and activation of NF-κB. These results may help in the design of novel and selective PKC inhibitors for the treatment of many inflammatory and neurological diseases in which production of NO plays a pathogenic role.

## Background

Microglia are distributed throughout the central nervous system (CNS) as resting immunocompetent cells derived from a monocyte/macrophage lineage [1,2]. When activated, microglia protect neurons by clearing toxic cell debris and pathogens, and acting as antigen presenting cells to induce innate immune responses [3]. However, excessive activation of microglia can also release a variety of toxic factors including reactive oxygen species (ROS), reactive nitrogen species (RNS) and proinflammatory cytokines, which cause toxicity to the neighboring cells such as neurons and oligodendrocytes (OLs). A pathogenic role for nitric oxide has been implicated in many inflammatory and neurodegenerative diseases, including multiple sclerosis, stroke and traumatic brain injury [4-7]. Understanding the potential mechanisms that turn beneficial inflammatory responses into detrimental action is crucial for identifying therapeutic targets to intervene in self-sustained inflammatory cycles.

Nitric oxide (NO), generated from L-arginine by nitric oxide synthase (NOS), has been shown to be both a signaling and an effector molecule in diverse biological systems [8-10]. Among the three isoforms of NOS identified, neuronal NOS (nNOS) and endothelial NOS (eNOS) are Ca^2+ ^dependent [8-13], and inducible NOS (iNOS) functions in a Ca^2+^-independent manner [10,13]. Induction of iNOS occurs primarily in astrocytes and microglia in response to endotoxin or to proinflammatory cytokines, such as TNFα, IL-1β or IFNγ [14]. Using pharmacological inhibitors and molecular approaches, studies have shown that NO can react with superoxide to form peroxynitrite in reactive microglia causing toxicity to neurons and OLs [15,16]. Although it is known that activation of various transcription factors - such as STAT, NF-κB, AP-1, and C/ERP - can contribute to the production of NO [17-20], the signaling pathways regulating expression of iNOS and production of NO in the CNS are still not well understood.

Protein kinase C (PKC) is a family of serine/threonine kinases that regulate cellular responses elicited by hormones, neurotransmitters and growth factors [21]. Based on differences in sequence homology between these isozymes and their requirements for cofactors, the PKC family is divided into conventional PKCs (cPKC: α, β and γ), novel PKCs (nPKC: δ, ε, η and θ) and atypical PKCs (aPKCs: ζ and λ/ι) [22,23]. PKC isoforms are widely expressed in many cell types, including microglia/macrophages [24], and studies have shown that PKC activation is an important mediator of microglial activation [25,26]. PKC inhibitors reduce NO synthesis from IFN-γ-treated microglia and PKC δ is able to regulate NF-κB activation and iNOS expression in mouse peritoneal macrophages [27]. Because of the existence of various PKC isoforms and the ambiguity of action of PKC inhibitors, the role of specific PKC isoforms involved in the inflammatory response in microglia has not been elucidated. In this study we used murine microglial cell line BV-2 cells to examine the signaling pathways by which PKC activation leads to iNOS induction in LPS-activated microglia. Our results indicate that all PKC isoforms are expressed in BV-2 cells with a particularly high expression of nPKC. Although several PKC isoforms can mediate lipopolysaccharide- (LPS-) stimulated increases in iNOS expression, PKC δ and β are likely the major PKC isoforms responsible for PKC function in reactive microglia. Furthermore, we found that distinct mitogen activated protein kinases (MAPKs) are activated in response to specific PKC isoforms and result in iNOS induction. Elucidation of the signaling pathways mediated by the different PKC isoforms in iNOS expression in reactive microglia will facilitate the development of isoform-specific PKC inhibitors with the potential to avoid the side effects of pan-PKC inhibitors.

## Methods

### Materials

Fetal bovine serum (FBS) and Dulbecco's modified Eagle's medium (DMEM) were purchased from Invitrogen (Carlsbad, CA). The BV-2 cell line was a generous gift from Dr. Feng-Qiao Li, Cognosci Inc., NC. Bacterial LPS (Escherichia Coli O111:B4) was obtained from Sigma (St. Louis, MO). 2',7'-dichlorohydrofluorescein diacetate (DCF) was purchased from Molecular Probes, Inc. (Eugene, OR). Antibodies against phosphorylated and total p38, extracellular signal regulated kinase 1/2 (ERK1/2) and c-Jun N-terminal kinase (JNK) were purchased from Cell Signaling Technology (Danvers, MA). Anti-iNOS antibody was purchased from BD biosciences (San Diego, CA). PKC siRNAs were purchased from Santa Cruz Biotechnology (Santa Cruz, CA). Bisindolylmaleimide-1 (Bis-1), Rottlerin, GO6976, SB203580, SP600125 and U0126 were purchased from Calbiochem (Gibbstown, NJ). Transfection reagents were from Roche (Basel, Switzerland) and Luciferase assay kit was from Promega (Madison, WI).

### Cell culture

Immortalized murine microglial cells (BV-2) were cultured in 100 mm dishes in DMEM containing 5% FBS, 1% penicillin/streptomycin at 37°C in an incubator with a humidified atmosphere of 95% air and 5% CO_2._

### Quantitative real-time PCR and reverse transcriptase PCR analysis

Total RNA was isolated from cultured BV-2 cells using RNeasy Mini Kit (Qiagen, Valencia, CA) and cDNA synthesis from total RNA was performed using a ReveriAid First Strand cDNA synthesis kit (Fermentas, Glen Burnie, MD) using 1 µg total RNA and 1 μl oligo (dT)_18 _following the manufacturer's instructions. Quantitative real time PCR was conducted with cDNA as a template in a 7500 Real time PCR System using SYBR Green PCR master mix (Applied Biosystems, Foster city, CA). The primers for target genes are shown in table 1. All samples were run in triplicate for PCR amplification. Relative values for mRNA expression were determined from their optimized threshold cycle (C_T_) normalized against the C_T _value of an internal control gene, GAPDH, by using the comparative C_T _method (User Bulletin 7500, Applied Biosystems). To test for downregulation of PKC isoforms by specific PKC siRNA, total mRNA isolated from PKC siRNA or RISC-free siRNA-transfected BV-2 cells was used to synthesize cDNA as described above. One microliter of each cDNA, synthesized in a reverse transcriptase reaction, was used for PCR amplification in the presence of 1 U *Taq *DNA polymerase in Tag buffer, 0.2 mM each of dNTPs, and 1 μM of each primer. Each sample was amplified for different cycles according to the expression level of each gene in the cells. PKC α, β and θ were amplified for 32 cycles, PKC ε and η were amplified for 28 cycles, and PKC δ was amplified for 26 cycles. The PCR amplification reaction used a three-step program (30 sec at 95°C, 30 sec at 60°C, and 45 sec at 72°C). The PCR product was run on 1.5% agarose gels and visualized under UV light.

**Table 1:** Primer sequences of mouse PKC isoforms.

PKC isoform	Forward	Reverse
α	5'-c c c a t t c c a g a a g g a g a t g a-3'	5'-t t c c t g t c a g c a a g c a t c a c-3'

β	5'-t c c c t g a t c c c a a a a g t g a g-3'	5'-a a c t t g a a c c a g c c a t c c a c-3'

δ	5'-c a g a c c a a g g a c c a c c t g t t-3'	5'-g c a t a a a a c g t a g c c c g g t a-3'

γ	5'-a c c a g g g c a t c a t c t a c a g g-3'	5'-c t t c c t c a t c t t c c c c a t c a-3'

ε	5'-g a g g a c t g g a t t g a c c t g g a-3'	5'-a t c t c t g c a g t g g g a g c a g t-3'

η	5'-c a t c c c a c a c a a g t t c a a c g-3'	5'-a t a t t t c c g g g t t g g a g a c c-3'

λ	5'-t a t g g c t t c a g c g t t g a c t g-3'	5'-c c t t t g g g t c c t t g t t g a g a-3'

θ	5'-a t g g a c a a c c c c t t c t a c c c-3'	5'-g c g g a t g t c t c c t c t c a c t c-3'

ζ	5'-a a g t g g g t g g a c a g t g a a g g-3'	5'-c a g c t t c c t c c a t c t t c t g g-3'

### PKC activity assay

The activity of PKC in BV-2 cells following LPS treatment was measured using a PKC activity assay kit from Assay Designs, Inc (Ann Arbor, MI). In brief, BV-2 cells cultured in 24-well plates were treated with 1 μg/ml LPS for 30 min and then washed with cold PBS twice and lysed with protein lysis buffer. Whole cell lysates were adjusted to equal protein concentrations with lysis buffer and the same volume of each sample was added to ELISA plates pre-coated with crebtide, a substrate that can be readily phosphorylated by PKC. ATP was added to each well to initiate reaction at 30°C for 90 min. After emptying the contents of each well, phosphospecific substrate antibody was added and incubated for 1 hr. The phosphorylated crebtide was quantitated following the manufacturer's instructions.

### Western blot analysis

Whole cell lysates from cultured BV-2 cells were obtained by using ice-cold protein lysis buffer (containing 1 × TBS, 1% Nonidet P-40, 0.5% sodium deoxycholate, 0.1% SDS, 0.004% sodium azide) with freshly added protease inhibitor cocktail and glycerophosphate and sodium orthovanadate. The lysates were subjected to centrifugation at 10,000 g for 10 min at 4°C. 5 μg of whole cell lysates were boiled for 5 min, and separated on Novex 4-12% Bis-Tris gel. Proteins were transferred to PVDF membrane using a Bio-Rad mini-trans-blot cell. Transferred blots were blocked by incubating the membranes with 5% BSA for 1 hr at room temperature to reduce non-specific binding. Blocked membranes were incubated with primary antibodies overnight. These antibodies include rabbit polyclonal anti-phosphorylated and total ERK1/2, JNK and p38 (all with dilution of 1:1000), mouse anti-iNOS (1:1000), mouse anti-PKC α, β, δ, ε and γ (BD Transduction Laboratories, 1:1000) and rabbit anti-PKC η, λ, θ and ζ polyclonal antibodies (Santa Cruz, all with 1:500 dilution). After washing with 1 × TBS-T (Tris-buffered saline containing 1% Tween 20), the membranes were incubated with goat anti-rabbit or goat anti-mouse horseradish peroxidase (HRP) conjugated secondary antibody (1:2000) for 1 hr at room temperature. Finally, the membranes were incubated in Chemiluminescence western blot detection reagents from Pierce (Rockford, IL) for 1 min and protein was visualized with Image Reader LAS-3000 software.

### Nitrite measurement

The level of accumulated nitrite in the medium was determined by the Greiss reaction. Briefly, 50 μl of Greiss reagent (3.9 mM N-(1-naphthyl)ethylenediamine/58 mM sulfanilamide/5% phosphoric acid) was added to 50 μl of culture supernatant in a 96-well plate. Absorbance was measured at wavelength 550 nm, and nitrite concentration was calculated from a standard curve of sodium nitrite.

### siRNA transfection

In order to specify the role of each PKC isoform in iNOS induction by LPS-activated microglia, double-stranded siRNA oligonucleotides for each PKC isoform (purchased from Santa Cruz) were transfected into BV-2 cells with X-treme transfection reagent (60 nmol siRNA/well). The day before the transfection, BV-2 cells were split and plated into 24-well plates at a density of 2 × 10^5 ^cells/well to assure cells around 80% confluency at the time of transfection. The transfected cells were continuously incubated at 37°C for 48 hr before use for further experiments. siGLO RISC-free siRNA from Dharmacon was used as a negative control and its fluorescence was also used for evaluating transfection efficiency.

### Plasmid transfection and luciferase assay

The reporter gene with NF-κB promoter was transfected into BV-2 cells. In brief, the cells were trypsinized and plated into 96-well plates at a density of 5 × 10^4 ^cells/well. The transfection was performed with FuGene HD transfection reagent. One microgram plasmid containing NF-κB promoter or GFP was mixed with 0.25 μl FuGene HD in a total volume of 5 μl of serum-free DMEM for each reaction. At 24 hr after transfection, cells were treated with LPS for 3 hr in the presence of various PKC and MAPK inhibitors. Assessment of luciferase activity in transfected cells was carried out with a luciferase reporter assay system from Promega following the manufacturer's instructions.

### Statistical analysis

Data were analyzed for statistical significance using a two-tailed t test (comparison of two data sets) or with analysis of variance (ANOVA, comparison of multiple data sets). A significant difference was determined as p < 0.05. All experiments were performed in triplicate and have been repeated at least three times.

## Results

### ALL PKC isoforms are present in microglia and activated by LPS

**Figure 1:**
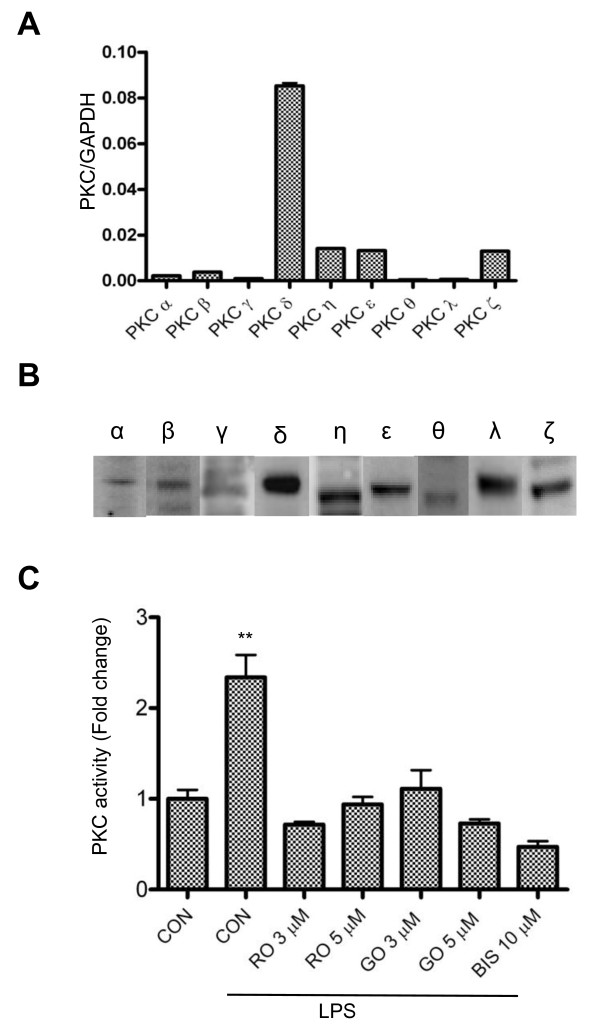
**Expression and activation of various PKC isoforms in BV-2 cells**. *A*, RNA samples isolated from the BV-2 cells were analyzed by quantitative real-time PCR, using primer pairs (see table 1) specific for mouse PKC α, β, γ, δ, ε, θ, η, ζ and λ. GAPDH was used as an internal control. A representative experiment of three that were performed is shown. *B*, Twenty micrograms of whole cell lysate from BV-2 cells for the detection of each PKC isoform was subjected to western blots with antibodies against PKC α, β, γ, δ, ε, θ, η, ζ and λ. A representative experiment of three that were performed is shown. *C*, BV-2 cells were treated with LPS (1 μg/ml) in the absence or presence of PKC inhibitors for 30 min and then lysed for assaying the PKC activity. CON, RO, GO and BIS stand for control, rottlerin, GO6976 and bisindolylmaleimide-1, respectively. All the PKC inhibitors completely blocked LPS-induced increases in PKC activity in BV-2 cells. **, p < 0.01 were obtained when the LPS alone-treated group was compared to the control and the drug-treated groups. A representative experiment of three that were performed is shown.

It has been reported that inhibitors of PKC can reduce iNOS induction in reactive microglia [28-30]. However, the specific PKC isoforms that are involved are not known. In order to identify the specific PKC isoforms that are required for iNOS production, we first examined which PKC isoforms are expressed in BV-2 by quantitative real-time PCR. The results indicate that while mRNAs encoding all the PKC isoforms are detectable, there are significantly higher levels of nPKC (δ, η, ε) expression compared to the conventional (α, β, γ) and the atypical (λ/ι) isoforms (Figure 1A). Using isoform-specific antibodies, we found that each of the PKC isoforms is also expressed in BV-2 cells (Figure 1B). In contrast to a report by Kang and colleagues [31], but consistent with results from Sun's group [29], we detected very low amounts of PKC α and β and very high levels of PKC δ, suggesting that nPKC isoforms may account for the major PKC activity in reactive microglia. In order to confirm PKC is activated in LPS-treated microglia, we measured PKC activity in murine BV-2 cells using ELISA. As shown in Figure 1C, PKC activity is elevated after treatment with LPS for 30 min, and suppressed by several PKC inhibitors, which include the pan-PKC inhibitor, Bis-1, the nPKC-selective inhibitor, rottlerin, and the cPKC-selective inhibitor, GO6976 [32,33]. These results demonstrate that both cPKC and nPKC might be functionally important in BV-2 cells when activated by LPS.

### PKC inhibitors attenuate iNOS expression in reactive microglia

**Figure 2:**
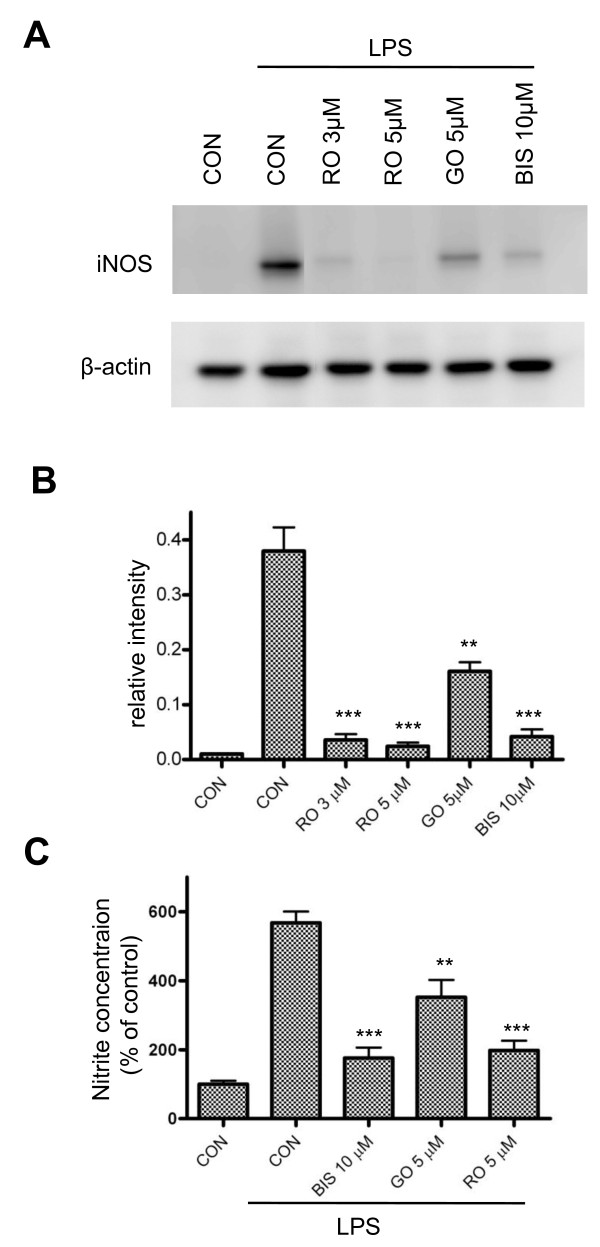
**Effects of PKC inhibitors on iNOS expression in BV-2 cells**. *A*, BV-2 cells were treated with LPS (1 μg/ml) for 6 hr in the presence of PKC inhibitors at the indicated concentrations. Aliquots of cell extracts prepared from the above treated cultures were subjected to western blot analysis by using antibodies against iNOS and β-actin. A representative experiment of four that were performed is shown. *B*, The levels of iNOS expression with various treatment conditions were normalized by β-actin and quantified using densitometric analysis. The data were pooled from 4 different experiments. **, p < 0.01 and ***, p < 0.001 were obtained when the GO, RO and BIS treated groups were compared to the LPS alone group. *C*. At 24 hr following LPS (1 μg/ml) treatment in the absence or presence of PKC inhibitors, culture media from 24-well plates were collected for measurement of nitrite production by reactive microglia. **, p < 0.01 and ***, p < 0.001 were obtained when the GO-, RO- and BIS-treated groups were compared to the LPS alone group. A representative experiment of four that were performed is shown.

The discovery of relatively isozyme-specific PKC inhibitors has provided important information regarding the function of individual PKC isoforms. It has been reported that rottlerin specifically inhibits PKC δ while GO6976 mainly targets conventional PKC, and Bis-1 has inhibitory effects on all PKC isozymes [32,33]. To determine whether iNOS induction is attributable to the activation of PKC, BV-2 cells were treated with LPS in the presence of the aforementioned PKC inhibitors. At 6 hr following LPS treatment, cells were lysed and iNOS production was determined by western blot. All of the PKC inhibitors were able to suppress iNOS expression to different degrees. However, rottlerin seems to have the greatest inhibitory effect (Figure 2A). In comparing these, rottlerin at 5 μM near completely (94%) blocks LPS-induced iNOS production, GO6976 at 5 μM causes 60% inhibition and Bis-1 at 10 μM inhibits iNOS production by 89% (Figure 2B). Consistently, NO production was also significantly attenuated when cells were treated with PKC inhibitors (Figure 2C). These results confirm that PKC activation is an integral component of LPS-induced iNOS expression and suggest that nPKC isoforms might play a prominent role in iNOS induction in BV-2 cells.

### Activation of MAPK occurs downstream PKC, but upstream iNOS induction in reactive microglia

**Figure 3:**
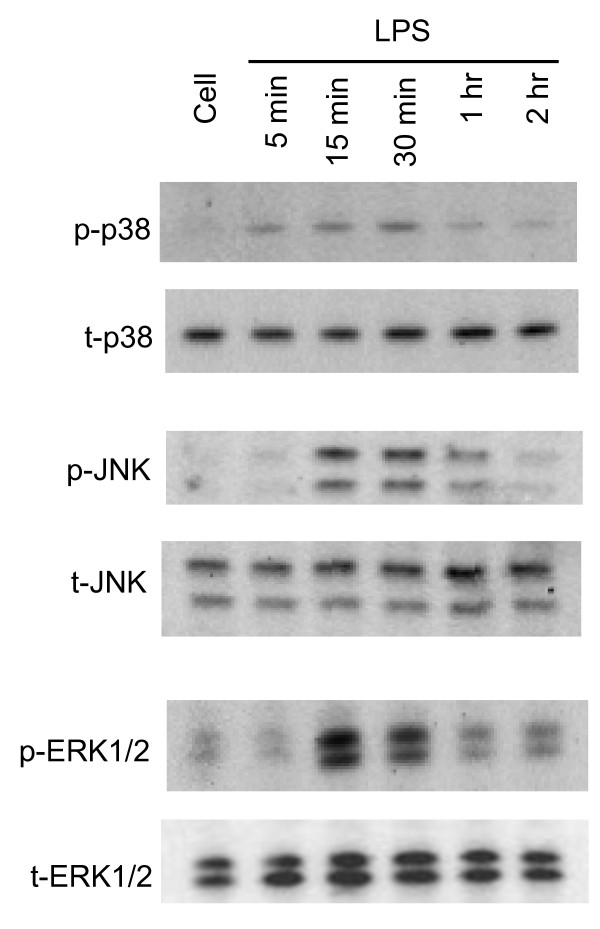
**Time course of MAPK activation induced by LPS in BV-2 cells**. BV-2 cells were treated with LPS (1 μg/ml) for 5, 15, 30 min, 1 or 2 hr and whole cell lysates at different time points were collected and subjected to western blot with antibodies against phosphorylated and total p38, JNK and ERK1/2, respectively. Phosphorylation of p38 occurred at 5 min following LPS treatment, whereas phosphorylation of ERK1/2 and JNK was observed after 15 min of LPS treatment. The phosphorylation of all three MAPKs started to diminish after 1 hr of LPS treatment. A representative experiment of three that were performed is shown.

It is well known that MAPK cascades are involved in cytokine- and LPS-mediated iNOS induction in microglial cells [34]. However, the involvement of specific MAPKs varies in different cell types and in response to different stimuli. At various times after LPS treatment, all three MAPKs in BV-2 cells are transiently phosphorylated. p38 phosphorylation occurs at 5 min, reaches maximum at 30 min, and nearly disappears at 1 hr following LPS treatment. The phosphorylation of JNK and ERK1/2 is present after 15 min of LPS treatment and remains at the same level until 30 min, followed by a dramatic reduction at 1 hr (Figure 3).

**Figure 4:**
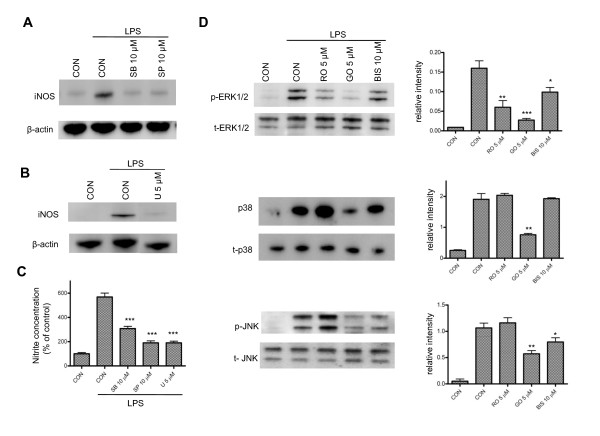
**Involvement of MAPK pathways in LPS-induced iNOS production in BV-2 cells**. *A-B*, BV-2 cells were treated with LPS (1 μg/ml) for 6 hr in the presence of SB205380 (SB), SP600125 (SP) or U0126 (U); inhibitors to block phosphorylation of p38, JNK (A) and ERK1/2 (B), respectively. The three MAPK inhibitors almost completely blocked iNOS induction in LPS-activated microglia. A representative experiment of three that were performed is shown. *C*. BV-2 cells were treated with MAPK inhibitors followed by LPS induction for 24 hr. Culture media were collected for measurements of nitrite production using a nitrite/nitrate assay kit. ***, p < 0.001 was obtained when the SB-, SP- and U-treated groups were compared to the LPS alone group. A representative experiment of four that were performed is shown. *D*, BV-2 cells were treated with LPS (1 μg/ml) for 15 min in the presence of PKC inhibitors at the indicated concentrations. Cell extracts from the treated cells were subjected to western blot with antibodies against phosphorylated and total ERK1/2, p38 and JNK. The relative intensities of phosphorylated versus total ERK1/2, p38 and JNK were quantified using densitometric analysis based on 3 different experiments. *, p < 0.05 and **, p < 0.01 were obtained when the drug-treated groups were compared to the LPS alone group.

Using U0126, SB203580 and SP600125, inhibitors of ERK1/2, p38 and JNK, respectively [35], we found that iNOS induction and NO production in reactive microglia were significantly inhibited (Figure 4A-C). There was no change in cell viability at 24 hr following drug treatment (data not shown). To investigate the possible relationship between PKCs and MAPKs, we examined activation of MAPKs in the presence of PKC inhibitors. We found that MAPK phosphorylation at 15 min following LPS treatment is attenuated by PKC inhibitors, indicating that activation of PKC occurs upstream of MAPKs. The nPKC selective inhibitor rottlerin attenuates ERK1/2 phosphorylation by 63%, but has no effect on the phosphorylation of p38 and JNK (Figure 4D). GO6976, a cPKC selective inhibitor, not only attenuates the phosphorylation of ERK1/2 by 83%, but also suppresses the phosphorylation of p38 and JNK by 60% and 47%, respectively (Figure 4D). The general PKC inhibitor, Bis-1, inhibits phosphorylation of ERK1/2 by 40% and JNK by 30%. Taken together, these results suggest that although all of the MAPKs are involved in induction of iNOS in LPS-treated microglia, activation of specific PKC isoforms may lead to phosphorylation of distinct MAPKs.

### Activation of NF-κB contributes to PKC-mediated iNOS induction in reactive microglia

**Figure 5:**
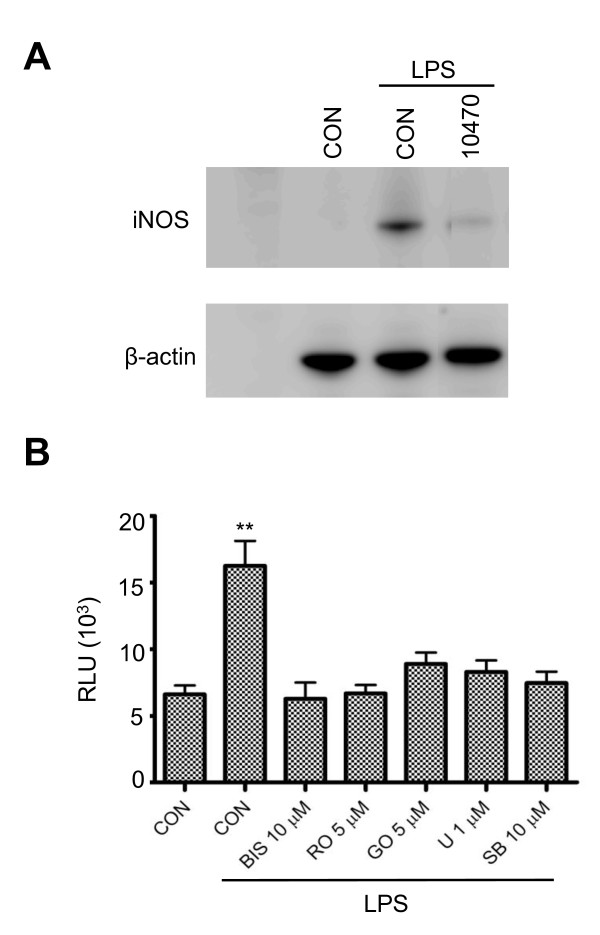
**Involvement of the NF-κB pathway in LPS-activated BV-2 cells**. *A*, BV-2 cells in 24-well plates were treated with LPS (1 μg/ml) for 6 hr in the presence of CAY10470 (20 nM), an NF-κB inhibitor. Cell lysates were subjected to western blot analysis with antibodies against iNOS and β-actin. A representative experiment of three that were performed is shown. *B*, BV-2 cells in 96-well plates were transiently transfected with Luc-reporter gene with NF-κB promoter for 24 hr and then treated with LPS (1 μg/ml) for 3 hr in the presence of PKC or MAPK inhibitors. Luminescence from the cells was evaluated with a luciferase reporter assay system. RLUs (relative luminescence units) reflect the activity of NF-κB. U stands for U0126, and SB stands for SB203580. **, p < 0.01 were obtained when the drug-treated groups were compared to the LPS alone group. A representative experiment of four that were performed is shown.

NF-κB is one of the primary transcription factors that regulates iNOS expression. The regulation of iNOS mediated by ERK1/2 and p38 MAPK has been shown to require NF-κB activation in rat glial cells [34,36]. In this study, we also investigated whether NF-κB is involved in PKC-mediated iNOS production. CAY10470 is a recently developed NF-κB inhibitor. It is synthesized from quinazoline derivative 6a, containing 4-phenoxyphenethyl moiety at the C(4)-position with an IC_50 _of 11 nM to inhibit NF-κB activation in human Jurkat cells [37]. CAY10470 significantly reduces iNOS production (Figure 5A), implying the involvement of NF-κB activation in iNOS production induced by LPS in BV-2 cells. To further examine the interaction of PKC activation and NF-κB during LPS treatment, we transfected BV-2 cells with an NF-κB-responsive luciferase construct containing an NF-κB response element and luciferase. This construct encodes the firefly luciferase reporter gene under the control of a minimal CMV promoter and tandem repeats of the NF-κB transcriptional response element (TRE). The NF-κB reporter (Luc) can easily and rapidly monitor NF-κB activity in the cells. Our data demonstrate that luciferase activity induced by LPS is significantly inhibited in the presence of the PKC inhibitors, rottlerin, GO6976 and Bis-1 (Figure 5B). Similarly, U0126 and SB203580 also significantly repress NF-κB activity (Figure 5B). Taken together, these results indicate that NF-κB acts downstream of PKC and MAPKs to transcriptionally regulate iNOS production.

### The differential role of PKC isoforms in LPS-induced iNOS production and MAPK activation in BV-2 cells

**Figure 6:**
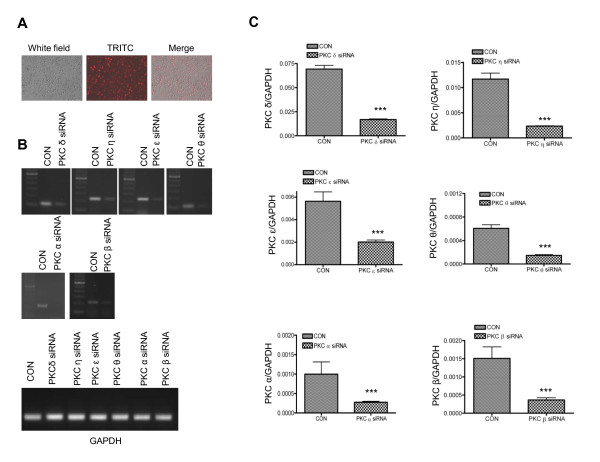
**Downregulation of PKC isoforms by specific PKC siRNA**. *A*, BV-2 cells transfected with DY-547 labeled RISC-free siRNA in 24-well plate were photographed using a Nikon Eclipse TE2000-U. The white field shows total cells, TRITC represents positively transfected cells, and merge stands for overlaid images. *B*, BV-2 cells were transfected with individual PKC isoform-specific siRNAs for 48 hr. Total RNAs were isolated from each transfected group for analysis of gene expression by reverse transcriptase PCR using primer pairs specific for PKC δ, ε, θ, η, α, β and GAPDH. The products were run on a 1% agarose gel impregnated with ethidium bromide. The bands were visualized under UV light. In each transfection panel, starting from the left, the lanes stand for DNA marker, RISC-free control siRNA and specific PKC siRNA, respectively. GAPDH was used as a loading control. *C*, Total mRNA was extracted from BV-2 cells that were transfected with PKC isoform siRNA or RISC free control siRNA (CON) for 48 hr and subjected to cDNA synthesis. The correspondent cDNA was then used as template for quantitative real time PCR with SYBR Green PCR Master mix. ***, p < 0.001 were obtained when the PKC siRNA transfected groups were compared to the RISC-free siRNA control group. A representative experiment of three that were performed is shown.

The above results suggest that LPS-induced iNOS production is mediated by PKC activation and MAPK phosphorylation. However, because of the lack of specificity and the potential non-target effects of the pharmacological inhibitors, it is still unclear whether specific PKC isoforms mediate microglial activation by LPS. To test this, we employed RNAi technologies to transfect BV-2 cells with isoform-specific siRNAs to suppress the expression of various PKC isoforms. To test for transfection efficiency, we used siGLO RISC-free siRNA as a positive control. siGLO RISC-free siRNA is a stable, fluorescent, and non-targeting control siRNA with RISC-free modification. Following 48 hr of transfection, at least 90% of cells were transfected (Figure 6A). The transfection efficiency was further demonstrated by downregulation of various PKC isoforms (PKCα, β, θ, δ, ε and η) using PKC isoform-specific siRNAs by both conventional and quantitative real-time PCR analysis (Figures 6B and 6C). qRT-PCR data indicated that specific PKC siRNA downregulates relative PKC isoform mRNA level by 3-5 fold.

**Figure 7:**
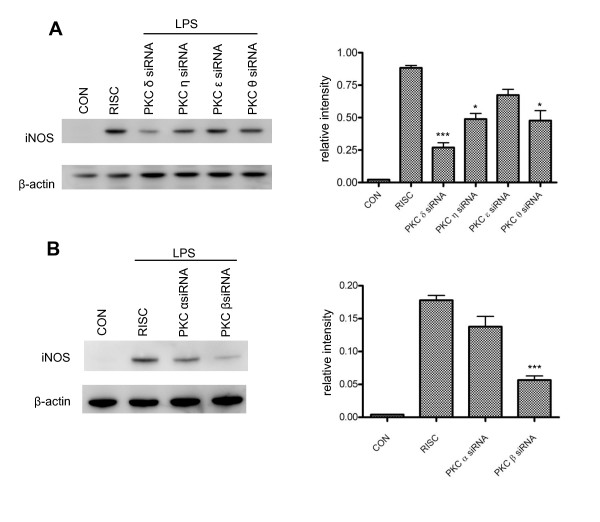
**Effects of downregulation of PKC isoforms on iNOS expression in LPS-activated BV-2 cells**. *A*, At 48 hr following nPKC siRNA transfection, cells were treated with LPS (1 μg/ml) for 6 hr and then lysed for western blot analysis. The ratio of relative band intensity of iNOS to β-actin from 4 different experiments is shown. *, p < 0.05 and ***, p < 0.001 were obtained when the PKC η, θ and δ siRNA transfected groups were compared to the RISC-free siRNA control group. *B*, At 48 hr following cPKC siRNA transfection, cells were treated with LPS (1 μg/ml) for 6 hr and then lysed for western blot analysis. The ratio of relative band intensity of iNOS to β-actin from 3 different experiments is shown. ***, p < 0.001 was obtained when the PKC β siRNA transfected group was compared to the RISC-free siRNA control group.

We then examined how downregulation of each specific PKC isoform could affect iNOS induction in BV-2 cells. At 48 hr following PKC siRNA transfection, cells were treated with LPS for 6 hr and iNOS expression was assessed by western blot (Figure 7A, B). Among the nPKC isoforms, knockdown of PKC δ appears to have the greatest inhibitory effect on iNOS expression, with a more-than-3-fold reduction observed. PKC η and θ knockdown reduces iNOS by almost 2-fold, and knockdown of PKC ε shows little effect (Figure 7A). Interestingly, downregulation of PKC β, but not PKC α, significantly attenuates iNOS induction (Figure 7B), even though a very low mRNA expression of both cPKC isoforms is observed in BV-2 cells (Figure 1A, B). There is a 3-fold reduction in iNOS expression in PKC β siRNA-transfected cells when compared to RISC-free siRNA-transfected controls (Figure 7B). In summary, these data demonstrate that each PKC isoform has a different potency in triggering iNOS induction in LPS-activated microglia and that selective inhibition of PKC δ or β may provide more focused anti-inflammatory effects.

**Figure 8:**
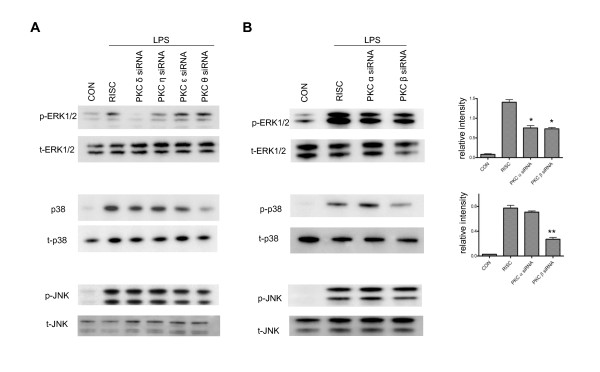
**Effects of downregulation of PKC isoforms on the phosphorylation of MAPK in LPS-treated BV-2 cells**. *A*, At 48 hr following PKC δ, η, ε or θ siRNA transfection, cells were treated with LPS (1 μg/ml) for 15 min and then lysed for western blot using antibodies against phosphorylated and total ERK1/2, p38 and JNK. A representative experiment of 3 that were performed is shown. *B*, At 48 hr following PKC α or β siRNA transfection, cells were treated with LPS (1 μg/ml) for 15 min and then lysed for western blot using antibodies against phosphorylated and total ERK1/2, p38 and JNK. *, p < 0.05 and **, p < 0.01 were obtained when the PKC siRNA transfection groups were compared to the RISC-free siRNA control group. The ratio of relative band intensity of phosphorylated ERK1/2 to total ERK1/2 and phosphorylated p38 to total p38 from 3 different experiments is shown.

To further identify the specific MAPK pathway through which PKC regulates the expression of iNOS, we examined the effect of PKC siRNAs on phosphorylation of various MAPKs. Similar to the results obtained using PKC inhibitors (Figure 4), downregulation of nPKCs produces various degrees of inhibition of the phosphorylation of ERK1/2 (Figure 8A). Knockdown of PKC δ almost completely blocks ERK1/2 activation. PKC η siRNA is shown to inhibit ERK1/2 phosphorylation by 60%, but PKC ε and θ siRNAs have no effect. Interestingly, PKC θ siRNA causes a 75% reduction of phosphorylation of p38 in LPS-treated microglia (Figure 8A), even though rottlerin doesn't exhibit any inhibitory effect (Figure 4C). Compared to the results obtained by using the cPKC inhibitor GO6976 (Figure 3C), we found that PKC β, but not PKC α siRNA, efficiently blocks phosphorylation of p38 by 65% based on densitometric analysis of the relative intensity of western blot bands (Figure 8B). However, both PKC α and β siRNAs display nearly 50% inhibitory effects on ERK1/2 phosphorylation (Figure 8B). In addition, the isoform-specific PKC siRNAs do not affect phosphorylation of JNK (Figure 8A, B), suggesting JNK activation is not involved in iNOS induction downstream of PKC activation. These results not only suggest that various PKC isoforms control diverse downstream MAPKs pathways to affect LPS-induced iNOS production in murine microglia, but also further demonstrate that the commonly used PKC inhibitors are less selective and that the use of individual PKC siRNAs should be more suitable for elucidating signaling pathways mediated by the various PKCs.

## Discussion

Overproduction of NO by enhanced iNOS induction has been tightly linked to neuroinflammatory and neurodegenerative diseases [38-40]. A better understanding of the signaling mechanisms involved in the regulation of microglial iNOS has potential therapeutic implications. Previous studies mostly used PKC activators and inhibitors to determine the role of PKC in the regulation of iNOS production in murine microglia [26-30]. However, the absence of selectivity and the potential off-target effects of these pharmacological agents limit the ability to further define isoform-specific functions of the various PKCs. In the present study, we have employed PKC isoform-specific siRNAs to delineate novel molecular signaling pathways linking PKC to iNOS induction in BV-2 cells when exposed to LPS.

### Role of the PKC specific isoforms in LPS-induced iNOS production

The PKC family consists of at least 10 serine/threonine protein kinases originally characterized by their dependency on lipids for catalytic activity [41,42]. The cPKCs require DAG and Ca^2+^, the nPKCs require DAG but not Ca^2+^, while the aPKCs require neither. The different modes of PKC regulation suggest that PKC isoforms may function differently in response to various stimuli. In BV-2 cells, pharmacological inhibition studies suggest that the nPKC and cPKC isoforms are integral to LPS-induced increases in iNOS expression and NO production (Figure 2), and isoform-specific siRNA knockdown confirms that PKC δ and PKC β are the major nPKC and cPKC isoforms involved in the regulation of LPS-induced iNOS production in murine microglia (Figure 7).

A number of studies have reported that particular PKC isoforms are involved in the production of NO in several different cell types [28,43-45]. Here we demonstrate a principal role for PKC δ and PKC β in the response to LPS exposure in murine BV-2 cells. These results are not only consistent with previous studies showing that PKC δ activation is required for regulating the production of iNOS in mouse peritoneal macrophages [27], human leukemia cells [46] and BV-2 cells [29], but also for the first time suggest that PKC β might play an important role in LPS-induced iNOS production in BV-2 cells even with its low levels of expression. It might be concluded that the primary role of PKC δ results from its high expression relative to other PKC isoforms (Figures 1A and 1B). However, PKC β expression is relatively low (Figure 1A) suggesting that induction of iNOS is dependent not only on levels of expression, but also on the activation of distinct PKC isoforms. Interestingly, PKC α and ε have been shown to be the major PKC isoforms involved in the signaling pathways by which IFNγ induces iNOS expression in the same cell line [28]. Collectively, these results suggest that distinct PKC isoforms are activated and implicated in the regulation of iNOS induction in a stimulus-specific manner.

### Downstream components of PKC activation in LPS-induced iNOS expression

*MAPKs*. In the present study we also explored signaling pathways downstream of PKC that increase iNOS expression in response to LPS exposure. In general agreement with the observed effects of the three PKC inhibitors, rottlerin, GO6976, and Bis-1 (Figure 4D), knockdown of PKC δ, η, α and β expression reduces LPS-induced phosphorylation of ERK1/2 (Figures 8A and 8B), whereas downregulation of PKC β significantly inhibits LPS-induced phosphorylation of p38 (Figure 8B). No effect on phosphorylation of JNK is observed with individual cPKC or nPKC siRNA (Figures 8A and 8B). Taken together, these results provide strong evidence that ERK1/2 and p38 are the main signaling pathways through which distinct PKC isoforms regulate iNOS induction in response to LPS. Moreover, these results suggest that distinct MAPKs are activated by specific PKC isoforms.

It has been shown that both p38 and ERK1/2 can mediate iNOS expression in glial cells [36]. However, the phosphorylation of ERK1/2 has been found to be involved in IFNγ-, but not in LPS-induced NO production, although NO production seems to be coupled to PKC δ activation under both stimulations [29]. The discrepancy between this report and our current study is unclear, but may be attributable to differences in the stage of BV-2 cells used in these studies. The same group has recently found that paraquat toxicity to microglia is mediated by PKC δ- and ERK1/2- dependent ROS generation [47]. The fact that neither nPKCs nor cPKCs affect JNK phosphorylation (Figure 6) suggests that JNK is not involved in the signaling pathway of iNOS induction coupling to PKC activation. Interestingly, PKC θ siRNA significantly blocks p38 phosphorylation (Figure 8A), although the commonly used nPKC inhibitor rottlerin has no inhibitory effect (Figure 4D). Similarly, GO6976 blocks JNK activation (Figure 4D) but the same phenomenon is not observed with the use of cPKC siRNAs (Figure 8B). These results further suggest that it might be misleading to draw conclusions on the role of specific PKC isoforms in the function of reactive microglia on the basis of pharmacological inhibition.

*NF-κB*. It is known that iNOS expression is transcriptionally regulated. Activation of p38 has been shown to regulate NF-κB, C/EBP, and ATF-2 to induce iNOS expression in rat astroglia [48]. However, HIV-1 Tat-induced iNOS expression in human astrocytes is dependent on phosphorylation of ERK1/2 and transcriptional activation of C/EBP, but not NF-κB [49]. These studies indicate that different transcription factors can be recruited via one or more kinase pathways with respect to different inducers of iNOS. In this study, we find that activation of NF-κB is required for iNOS induction through the application of CAY10470, an NF-κB-specific inhibitor (Figure 5). The observation that all of the PKC inhibitors - GO6976, rottlerin and Bis-1 - significantly block NF-κB activation strongly supports the conclusion that NF-κB activation is required for iNOS induction in LPS-treated BV-2 cells.

## Conclusions

**Figure 9:**
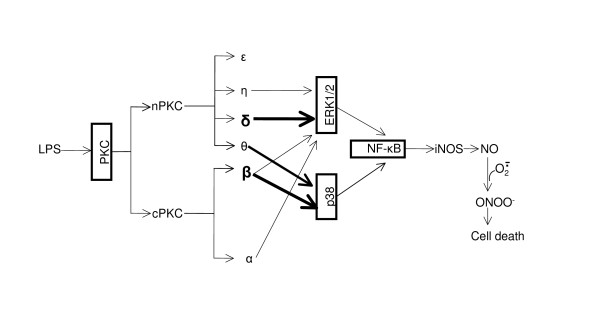
**Novel pathway of PKC and MAPK-mediated transcriptional activation of iNOS in LPS-treated BV-2 cells**. LPS activates cPKC and nPKC isoforms in BV-2 cells. Activation of PKC δ, η, α and β leads to ERK1/2 phosphorylation, whereas activation of PKC β and θ mainly phosphorylates p38. Phosphorylation of ERK1/2 and p38 converges to the activation of NF-κB and results in upregulation of iNOS and production of NO. NO can then react with superoxide to form the potent oxidant, peroxynitrite, culminating in cell death. The heavy arrows indicate that PKC δ and β are the two principal PKC isoforms involved in the signaling pathway proposed.

By using pharmacological inhibitors and RNA interference, we have clearly demonstrated that LPS-induced iNOS expression and NO production in BV-2 is mediated by a signaling pathway involving the sequential activation of PKC, MAPK and NF-κB as illustrated in Figure 9. In addition to elucidating the critical role of PKC δ in ERK1/2 phosphorylation and iNOS induction, our study reveals that PKC β is also a principal PKC isoform triggering iNOS induction in reactive microglia, which is coupled through phosphorylation of p38. The partial inhibitory effects of PKC η and θ on iNOS induction are due to their attenuation of the phosphorylation of ERK1/2 and p38, respectively. These data suggest that a novel interaction between the distinct PKC isoforms and the various MAPKs promotes iNOS induction. This interaction in different cell types may help to explain the discrepancy in the literature, and may also help guide the design of novel and selective PKC inhibitors for the treatment of many inflammatory and neurological diseases in which overproduction of nitric oxide plays a pathogenic role.

## Competing interests

The authors declare that they have no competing interests.

## Authors' contributions

JW designed and performed the experiments, analyzed the data and drafted the manuscript. RR performed some experiments and data analysis. YZ conceived of the study, participated in its design and coordination and helped to draft the manuscript. All authors read and approved the final version of the manuscript.
